# Muscle-fiber transdifferentiation in an experimental model of respiratory chain myopathy

**DOI:** 10.1186/ar4076

**Published:** 2012-10-29

**Authors:** Nils Venhoff, Dirk Lebrecht, Dietmar Pfeifer, Ana C Venhoff, Emmanuel Bissé, Janbernd Kirschner, Ulrich A Walker

**Affiliations:** 1 Department of Rheumatology & Clinical Immunology, University Medical Center Freiburg, Hugstetter Str. 55, Freiburg, 79104, Germany; 2 Department of Hematology & Oncology, University Medical Center Freiburg, Hugstetter Str. 55, Freiburg, 79104, Germany; 3 Department of Clinical Chemistry, University Medical Center Freiburg, Hugstetter Str. 55, Freiburg, 79104, Germany; 4 Department of Neuropediatrics and Muscle Disorders, University Medical Center Freiburg, Hugstetter Str. 55, Freiburg, 79104, Germany; 5 Department of Rheumatology, Basel University, Burgfelderstr. 101, Basel, 4012, Switzerland

## Abstract

**Introduction:**

Skeletal muscle fiber composition and muscle energetics are not static and change in muscle disease. This study was performed to determine whether a mitochondrial myopathy is associated with adjustments in skeletal muscle fiber-type composition.

**Methods:**

Ten rats were treated with zidovudine, an antiretroviral nucleoside reverse transcriptase inhibitor that induces a myopathy by interfering with mitochondrial functions. Soleus muscles were examined after 21 weeks of treatment. Ten untreated rats served as controls.

**Results:**

Zidovudine induced a myopathy with mitochondrial DNA depletion, abnormalities in mitochondrial ultrastructure, and reduced cytochrome *c *oxidase activity. Mitochondrial DNA was disproportionally more diminished in type I compared with type II fibers, whereas atrophy predominated in type II fibers. Compared with those of controls, zidovudine-exposed soleus muscles contained an increased proportion (256%) of type II fibers, whereas neonatal myosin heavy chains remained repressed, indicating fiber-type transformation in the absence of regeneration. Microarray gene-expression analysis confirmed enhanced fast-fiber isoforms, repressed slow-fiber transcripts, and reduced neonatal fiber transcripts in the mitochondrial myopathy. Respiratory chain transcripts were diminished, whereas the enzymes of glycolysis and glycogenolysis were enhanced, indicating a metabolic adjustment from oxidative to glycolytic capacities. A coordinated regulation was found of transcription factors known to orchestrate type II fiber formation (upregulation of *MyoD, Six1, Six2, Eya1*, and *Sox6*, and downregulation of *myogenin *and *ERRγ*).

**Conclusions:**

The type I to type II fiber transformation in mitochondrial myopathy implicates mitochondrial function as a new regulator of skeletal muscle fiber type.

## Introduction

Low muscle endurance and fatigue are frequent symptoms of patients with diseases that limit the oxygen supply of muscles by its capillaries or muscular oxygen use by its mitochondria. Muscle capillaries are lost in dermatomyositis [1], systemic sclerosis [2-4], and chronic obstructive pulmonary disease (COPD) [5], and qualitative or quantitative defects of respiratory chain components are found in the mitochondrial myopathies [6,7]. Ultrastructural changes in mitochondria and respiratory-chain dysfunction can also be induced by medications (statins [8], zidovudine, and other antiretroviral nucleoside analogues [9], and potentially, alcohol [10]). The physiological explanations for muscle fatigue and the adjustments of muscle metabolism to such respiratory compromise have, however, been only poorly addressed.

In humans, most skeletal muscles are equipped with more than one fiber type to accommodate a wide range of forces, kinetics, and endurance. Muscles specialized for maintaining postural tone have a high proportion of fibers that contract slowly (type 1 fibers), whereas muscles specialized for rapid movements contain a high proportion of fast-twitch (type 2) fibers. To account for fiber-type diversity, virtually every contractile protein of muscle fibers exists in different isoforms. Muscle-fiber types have also developed fine-tuned systems of energy delivery, which result in diverse metabolic profiles and oxygen requirements. Fiber types are, however, not static, as endurance training, weight loading, or hormonal factors can promote fiber-type transformation, even in adult muscles, by means of a coordinated antithetic regulation of fast and slow gene programs [11-13]. No study has investigated skeletal muscle fiber-type adjustments in response to a primary defect of the mitochondrial respiratory chain.

We therefore investigated how skeletal muscles adjust to mitochondrial dysfunction and whether they can alter their fiber-type composition. In this study, we modeled a mitochondrial myopathy by feeding rats with zidovudine, a nucleoside-analogue reverse transcriptase inhibitor that impairs with the replication of mitochondrial DNA and interferes with mitochondrial function through a variety of mechanisms, including competition with the normal nucleotide triphosphates for incorporation into replicating mtDNA chains, impairment of chain elongation, and excision-repair steps (extensively reviewed elsewhere) [14]. On a global basis, zidovudine is widely used in the treatment of human immunodeficiency virus (HIV) infections and can also cause a myopathy in humans [9,15]. Our experiments are the first to describe the ability of skeletal muscle to change fiber-type composition by downregulating the proportion of slow fibers and upregulating fast fibers in response to mitochondrial dysfunction. The changes in fiber-type composition are accompanied by metabolic adjustments from oxidative to more glycolytic capacities.

## Materials and methods

### Animals

Male Wistar rats were purchased at Charles River (Sulzfeld, Germany), were fed a normal rat chow (SSniff R/M-H; Spezialdiäten, Soest, Germany) *ad libitum*, and were housed in a normal night-day rhythm under standard conditions of temperature and humidity. At 7 weeks of age, 10 rats received zidovudine (kindly provided by GlaxoSmithKline, Munich, Germany) in the drinking water (100 mg/kg/d). This daily dose of zidovudine corresponds to the human dosage adjusted for body area and the higher metabolic and drug-disposal rate of rodents and was calculated on the basis of a daily liquid consumption of 20 ml [16,17]. Control rats (*n *= 10) did not receive any zidovudine.

Observations for fluid consumption, clinical signs, and mortality were carried out daily; body weights were recorded weekly. All rats were killed by cervical dislocation at age 28 weeks, immediately before organ collection and postmortem examination. Soleus muscle was snap frozen and cryopreserved in liquid nitrogen until subsequent analysis. Muscle aliquots were fixed in glutaraldehyde (3%) for subsequent electron microscopy. Serum was collected by puncture of the *Venae saphenae laterales *[18] before cervical dislocation in anesthesia with isoflurane (Abbott, Wiesbaden, Germany). All animal work was performed after animal welfare board approval (Regierungspräsidium Freiburg; Department 3, Nr. 35/9185/.81/G-07/67) and conformed to institutional guidelines as well as to the NIH policy [19].

### Histopathology and mitochondrial ultrastructure

Soleus muscle-fiber diameters were morphometrically quantified in all rats on three randomly selected 0.09-mm^2 ^areas of 8 μm thick, hematoxylin and eosin-stained sections, by using an automated image-analysis and processing software (Leica QWin Standard v2.7; Leica Microsystems, Imaging Solutions, Cambridge, UK). The histochemical assay for myofibrillar ATPase activity (pH 4.35 or 10.5) was used to distinguish and morphometrically count fast and slow muscle fibers [20]. On 4-μm cryostat muscle transverse sections, succinate dehydrogenase (SDH) and cytochrome *c*-oxidase (COX) histochemistry was performed [21]. The evaluating person was blinded to the group status of all animals. Two randomly selected soleus muscle samples from each group were examined with electron microscopy, as described [22].

### Myosin heavy-chain immunohistochemistry

Fiber-type analyses were confirmed in muscle cryosections (8 μm) incubated overnight with 1:100 diluted antibodies against fast or slow myosin heavy-chain isoforms (clones WB-MHCF and WB-MHCS; Novocastra, Newcastle, UK). To determine signs of regenerating fibers, we used anti-neonatal myosin heavy-chain antibody (clone WB-MHCn; Novocastra). An Alexa-fluor 488 conjugated secondary antibody (IgG anti-mouse; Novacastra) was used. In images obtained from immunohistochemistry, muscle fiber-type composition was also quantified by using automated image analysis (Leica QWin Standard v2.7; Leica Microsystems). The evaluating person was blinded to the group status of the animals.

### Respiratory-chain enzyme activities

Histochemical COX and SDH staining is difficult to quantify reliably. We therefore measured the activities of COX, SDH, and nicotinamide adenine dinucleotide hydrogen dehydrogenase (NADH-DH) in freshly prepared soleus muscle extracts with spectrophotometric assays, as described [23]. NADH-DH and COX are the multisubunit complexes I and IV of the mitochondrial respiratory chain and are encoded partly by nuclear DNA (nDNA) and partly by mtDNA, whereas SDH is a respiratory chain component (complex II), which is encoded entirely by nDNA.

### Serum parameters

Serum concentrations of creatinine kinase, resting lactate, glucose, aspartate aminotransferase, alanine aminotransferase, and creatinine levels were determined photometrically by using a Roche/Hitachi 917/Modular P analyzer (Mannheim, Germany), according to the manufacturer's instructions.

### Single-fiber mtDNA copy numbers

In each animal, three fast and three slow fibers were picked with a microcapillary under an inverted microscope from a 14-μm-thick, ATPase activity (pH 10.5) typed, transverse soleus muscle section [24]. Total DNA from single fibers was released with 5 μl of a solution containing 200 m*M *KOH and 50 m*M *dithiothreitol (incubated for 1 hour at 65°C), followed by a neutralizing buffer (5 μl) containing 900 m*M *Tris-HCl, pH 8.3, and 200 m*M *HCl [24]. MtDNA and nDNA copy numbers were quantified from 2 μl of the solute by quantitative PCR, as described [25]. Amplifications of mitochondrial and nuclear products were performed in triplicate. Absolute mtDNA and nDNA copy numbers were calculated by using serial dilutions of plasmids with known copy numbers.

### Microarray analysis

RNA was extracted from eight randomly selected frozen muscles from each group with the Uneasy Kit (Qiagen, Hilden, Germany). Quantity and integrity of the RNA were verified by using RNA 6000 nano chips (2100 Bioanalyzer; Agilent, Palo Alto, CA, USA). RNA samples (500 ng) with an RNA integrity number of greater than 9 were further processed with the GeneChip Whole Transcript Sense Target Labelling Assay from Affymetrix (Santa Clara, CA, USA) according to the manufacturer's instructions.

Arrays were scanned with the Affymetrix GeneChip Scanner 3000 7G, and raw data were imported into the Refiner module of Genedata Expressionist software (Martinsried, Germany, version 5.3.5), in which quantile normalization and probe summarization was performed by using its Refiner condensing algorithm [26]. The microarray data were uploaded (ArrayExpress accession number: E-MEXP-3642) in the ArrayExpress Archive [27].

### Statistics

The Kolmogorov-Smirnov test was used to analyze for normal distribution. Groups were then compared with ANOVA, Mann-Whitney, unpaired *t *test, or Wilcoxon analysis, as appropriate. Skewed data are provided as median plus interquartile ranges (IQRs), and normally distributed data, as group means and standard deviation (SD). Correlations were computed as nonlinear exponential regressions. All graphics and calculations were performed by using the Sigma Plot 2000, version 8.0 (SPSS, Inc.) and the Sigma Stat, version 3.1 (Jandel Inc.) packages.

To identify differentially expressed genes between the groups in microarray analysis, the unpaired Bayes *T *test (CyberT) [26] with the Bayes confidence estimate value set to 24 and a window size of 101 genes, as well as 100% valid values in each group, was performed with the Analyst module of Expressionist. To estimate the false-discovery rate, the Benjamini-Hochberg *q *value was calculated in a sequential Bonferroni-type procedure [28]. We then used the "N-fold regulation" activity of Analyst to calculate the median ratio between the experimental groups. Only genes from the categories "main" and "unmapped" (see Affymetrix transcript annotation RaGene-1_0-st-v1.na30.1.rn4.transcript) were included, thereby omitting control probes or genes with uncertain annotation. The false-discovery rate, which estimates the number of false positives within a list of significant genes, was chosen as 10%.

## Results

### Zidovudine induces a respiratory-chain myopathy

**Figure 1:**
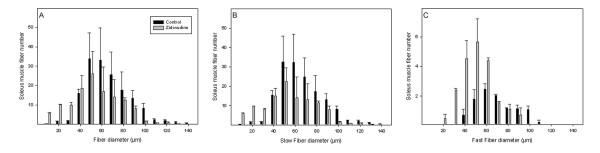
**Average numbers of soleus muscle fiber diameters (A)**. Fiber thinning in type I **(B) **and in type II fibers **(C)**. Values represent group means (±SD).

**Figure 2:**
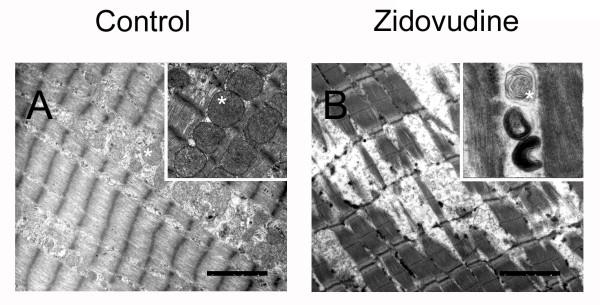
**Representative electron micrographs demonstrate zidovudine-induced degeneration of the myofibrillar lattice**. Abnormal mitochondria (star) with disrupted crystal architecture. Magnification bars, 2.5 μm.

The daily fluid consumption and body weight of the rats was unaffected by zidovudine (data not shown). The autopsy did not reveal macroscopic organ anomalies. Soleus muscle fiber diameters were decreased in the zidovudine group (Figure 1, Table 1). After 28 weeks, groups did not differ in serum levels of creatinine kinase, resting lactate, and glucose. Serum creatinine levels, however, were lower in rats treated with zidovudine, indicating reduced muscle mass (*P *= 0.001) compared with untreated rats. Electron microscopy revealed a focal disarray of the myofibrillar lattice in the zidovudine group (Figure 2). The crystal architecture was lost in a substantial proportion of the organelles and contained deposits of electron-dense material. Mean mtDNA copy numbers were decreased by 29% (*P *< 0.001) in zidovudine-treated rats compared with control animals (Table 1). Histochemical COX/SDH staining showed a uniformly downregulated respiratory-chain activity and no clear fiber type-specific pattern. NADH-DH and COX activities in the soleus muscle were depressed in the zidovudine group (*P *= 0.042 and *P *= 0.026, respectively; Table 1). In contrast, the activity of SDH was unaffected (*P *= 0.7; Table 1). These data indicate that zidovudine induced a metabolic myopathy with depleted mtDNA copies and a specific downregulation of mtDNA-encoded respiratory chain activities and consecutive fiber atrophy.

**Table 1:** Effects of zidovudine on soleus muscle histology, serum parameters, mtDNA, mitochondrial function, and muscle fiber-type characteristics

Complete soleus muscle	Control (*n *= 10)		Zidovudine (*n *= 10)		*P *value	
Soleus muscle fiber diameter (median μm)	58.3 (IQR 54.2, 62.3)		41.1 (IQR 37.5, 47.1)		<0.001	
Serum creatine kinase (U/L)	724 ± 480		548 ± 257		0.3	
Serum creatinine (mg/L)	3.6 ± 0.34		3.1 ± 0.11		<0.001	
Serum glucose (mg/L)	1,760 ± 100		1,680 ± 220		0.055	
Serum resting lactate (m*M*)	4.5 ± 1.8		5.0 ± 1.6		0.9	
Serum alanine aminotransferase (U/L)	66 ± 8		56 ± 7		0.1	
Serum aspartate aminotransferase (U/L)	125 ± 35		101 ± 13		0.09	
mtDNA copy number (mean copies/myonucleus)	559 ± 46		399 ± 78		<0.001	
NADH DH (μmoles min^-1 ^g muscle protein^-1 ^)	684 ± 254		474 ± 236		0.042	
COX (μmoles min^-1 ^g muscle protein^-1 ^)	28 ± 8		18 ± 9		0.026	
SDH (μmoles min^-1 ^g muscle protein^-1 ^)	36 ± 6		35 ± 10		0.7	
NADH DH/SDH-ratio (% of control mean)	100 ± 28		72 ± 20		0.027	
COX/SDH-ratio (% of control mean)	100 ± 27		67 ± 26		0.016	

Single-fiber soleus muscle	Control		Zidovudine		p-value	

Fiber type	Fast	Slow	Fast	Slow	Fast	Slow

Fiber-type proportion (% of all fibers)	5.2 ± 3.0	94.8 ± 3.0	13.3 ± 5.1	86.7 ± 5.1	<0.001	<0.001
Fiber diameter (mean μm)	54.8 ± 5.8	65.5 ± 10.6	37.3 ± 9.3	51.8 ± 6.2	<0.001	0.006
mtDNA copy number (mean copies/myonucleus)	617 ± 91	683 ± 94	384 ± 91	206 ± 97	<0.001	<0.001

Values represent group means (±SD); soleus muscle-fiber diameter values are given as median with interquartile range.

### Type II fibers are enhanced in mitochondrial myopathy

Muscle morphometry revealed that the fiber diameter was reduced in both fast and slow fibers of zidovudine-treated rats (Table 1), leading to an increased fiber number per microscopic area (151 ± 20 fibers/μm^2 ^in rats without versus 194 ± 26 fibers/μm^2 ^in rats with zidovudine; *P *= 0.002). Fast fibers, however, had a disproportionate degree of atrophy (32% reduction of mean fiber diameter; *P *< 0.001) compared with slow fibers (21% reduction; *P *= 0.006).

In control muscle, slow fibers and fast fibers contained similar numbers of mtDNA copies (*P *= 0.15). In zidovudine-treated rats, however, slow fibers had fewer mtDNA copies than did fast fibers (*P *= 0.002). Zidovudine treatment also induced a greater proportion of mtDNA depletion in slow fibers than in fast fibers (70% versus 38%; *P *< 0.001; Table 1).

**Figure 3:**
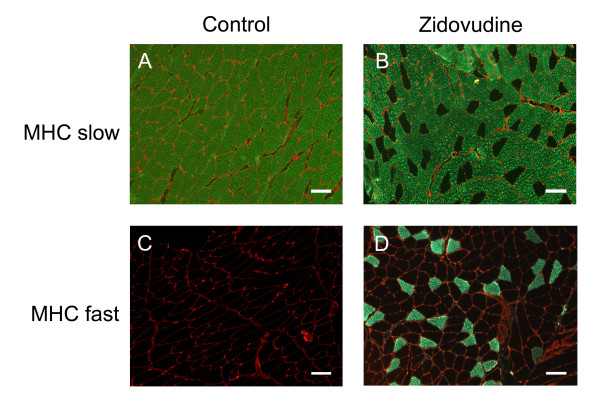
**Immunohistochemistry demonstrating increased numbers of type II (fast) fibers in zidovudine-exposed soleus muscle (magnification bars, 100 μm)**.

As expected, histomorphometry in ATPase-typed soleus muscle of control rats demonstrated the vast predominance of type 1 slow oxidative fibers (94.8% ± 3.0%) relative to fast glycolytic fibers. In contrast, zidovudine-treated animals, soleus muscles contained a high proportion of fast fibers (256% increase compared with controls; *P *< 0.001), whereas slow fibers were diminished (Table 1). Fiber-type grouping was not evident. The upregulation of fast fibers in soleus muscle exposed to zidovudine was confirmed with antibodies specific for fast and slow myosin heavy chain (Figure 3). Immunofluorescence studies of zidovudine-treated soleus muscles were carried out with an antibody directed against neonatal MHC. None of the soleus muscle fibers expressed the neonatal myosin heavy-chain isoform, indicating the absence of fiber regeneration (not shown). Furthermore, we did not observe signs of muscle denervation in terms of fiber-type grouping or upregulated neonatal myosin heavy chains (data not shown).

### Enhanced fast-fiber and repressed slow-fiber transcripts

**Figure 4:**
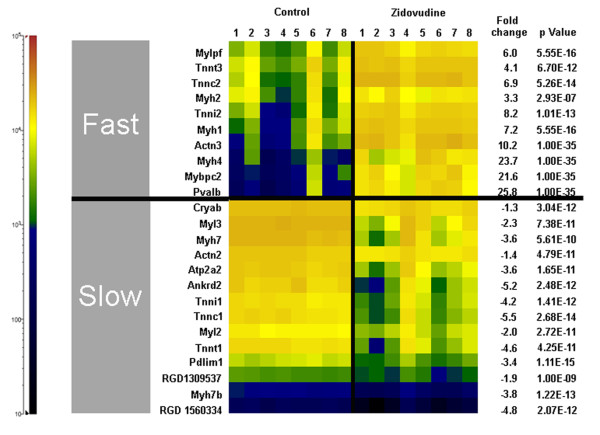
**Soleus muscle gene transcripts indicative of fast or slow myofiber type**. The cell color codes in the heat map indicate relative transcript amounts in the soleus muscles of eight control rats and eight rats treated with zidovudine. Fast myofiber transcripts: Mylpf, myosin light chain, phosphorylatable (skeletal fast); Tnnt3, troponin T type 3 (skeletal, fast); troponin C type 2 (skeletal fast); Myh2, myosin, heavy polypeptide 2 (skeletal muscle, adult); Tnni2, troponin I type 2 (skeletal, fast); Myh1, myosin, heavy polypeptide 1 (skeletal muscle, adult); Actn3, actinin α3; Myh4, myosin, heavy-chain 4, (skeletal muscle); Mybpc2, myosin-binding protein C, fast-type; Pvalb, parvalbumin. Slow myofiber transcripts: Cryab, crystallin, αB; Myl3, myosin, light-chain 3, alkali; (ventricular, skeletal, slow); Myh7, myosin, heavy-chain 7, cardiac muscle, β; Actn2, actinin α2; Atp2a2, ATPase, Ca^2+ ^transporting, cardiac muscle, slow twitch 2; Ankrd2, ankyrin repeat domain 23; Tnni1, troponin I type 1 (skeletal, slow); Tnnc1, troponin C type 1 (slow); Myl2, myosin, light polypeptide 2, regulatory, cardiac, slow; Tnnt1, troponin T type 1 (skeletal, slow); Pdlim1, PDZ and LIM domain 1; RGD1309537, similar to myosin regulatory light-chain 2-A, smooth muscle isoform (myosin RLC-A); Myh7b, myosin, heavy-chain 7B, cardiac muscle, β; RGD1560334, similar to myosin light-chain 1 slow α.

Microarray gene expression analysis identified 1,411 genes and 43 pathways to be significantly regulated (*P *< 0.01) in zidovudine-treated animals relative to control. In zidovudine-exposed rat soleus muscle, fast-fiber transcripts were significantly enhanced, and slow-fiber transcripts, repressed (Figure 4). Consistent with the absence of fiber regeneration in the immunofluorescence studies, embryonal and neonatal myosin heavy-chain transcripts were downregulated [29].

Investigating metabolic adjustments, we found nucleus- and mtDNA-encoded respiratory chain subunits to be coordinately downregulated in zidovudine myopathy, although many changes were not statistically significant. The transcription of the rate-limiting enzymes of glycolysis and glycogenolysis was enhanced (Table 2) and the mitochondrial carnitine shuttle (carnitine palmitoyltransferase, *CPT1b*), and β-oxidation (3-hydroxy-acyl-CoA dehydrogenase) downregulated.

**Table 2:** Microarray gene expression of soleus muscle-fiber energy metabolism and gene products involved in fiber-type regulation

Gene description (gene symbol)	FC	*P *value	*q *value
Respiratory chain complex I			

NADH dehydrogenase (ubiquinone) 1α subcomplex, 4 (Ndufa4)	-1.1	0.007	0.999
NADH dehydrogenase (ubiquinone) 1α/β subcomplex, 1 (Ndufab1)	1.0	0.507	0.999
NADH dehydrogenase (ubiquinone) 1α subcomplex, assembly factor 1 (Ndufaf1)	-1.1	0.110	0.999
NADH dehydrogenase (ubiquinone) 1β subcomplex 3 (Ndufb3)	-1.0	0.772	0.999
NADH dehydrogenase (ubiquinone) 1β subcomplex, 5 (Ndufb5)	-1.1	0.036	0.999
NADH dehydrogenase (ubiquinone) 1 β subcomplex, 9 (Ndufb9)	-1.0	0.286	0.999
NADH dehydrogenase (ubiquinone) 1, subcomplex unknown, 1 (Ndufc1)	-1.1	0.167	0.999
NADH dehydrogenase (ubiquinone) Fe-S protein 1 (Ndufs1)	-1.1	0.045	0.999
NADH dehydrogenase subunit 1 (mt-ND1)	-1.1	0.267	0.999
NADH dehydrogenase subunit 2 (mt-ND2)	-1.1	0.087	0.999
NADH dehydrogenase subunit 4L (mt-ND4l)	-1.1	0.004	0.999
NADH dehydrogenase subunit 5 (mt-ND5)	-1.1	0.002	0.999
NADH dehydrogenase subunit 6 (mt-ND6)	-1.2	0.364	0.999

Respiratory chain complex II			

Succinate dehydrogenase complex, subunit B, iron sulfur (Ip) (Sdhb)	-1.4	0.00001	0.139

Respiratory chain complex III			

Ubiquinol-cytochrome *c *reductase binding protein (Uqcrb)	-1.1	0.003	0.999
Ubiquinol-cytochrome *c *reductase core protein I (Uqcrc1)	-1.0	0.677	0.999
Ubiquinol cytochrome *c *reductase core protein 2 (Uqcrc2)	-1.1	0.039	0.999

Respiratory chain complex IV			

Cytochrome *c *oxidase, subunit Va (Cox5a)	-1.1	0.205	0.999
Cytochrome *c *oxidase subunit Vb (Cox5b)	-1.0	0.442	0.999
Cytochrome *c *oxidase, subunit VIa, polypeptide 2 (Cox6a2)	-1.1	0.096	0.999
Cytochrome *c *oxidase, subunit VIc (Cox6c)	-1.1	0.144	0.999
Cytochrome *c *oxidase subunit VIIb (Cox7b)	1.0	0.501	0.999
Cytochrome *c *oxidase, subunit VIIa 2 (Cox7a2)	-1.1	0.006	0.999
Cytochrome *c *oxidase subunit VIIa polypeptide 2 like (Cox7a2l)	-1.2	0.002	0.999
Cytochrome *c *oxidase subunit I (mt-Co1)	-1.1	0.000	0.999
Cytochrome *c *oxidase subunit II (mt-Co2)	-1.1	0.028	0.999

Respiratory chain complex V			

ATP synthase, F1 complex, γ polypeptide 1 (Atp5c1)	1.0	0.807	0.999
ATP synthase, F0 complex, subunit C3 (subunit 9) (Atp5g3)	-1.1	0.080	0.999
ATP synthase GA binding protein transcription factor, α subunit: (Gabpa: Atp5j)	-1.2	0.005	0.999
ATP synthase, F0 complex, subunit F2 (Atp5j2)	1.0	0.548	0.999
ATP synthase F0 subunit 6 (mt-atp6)	-1.3	0.739	0.999
ATP synthase F0 subunit 8 (mt-atp8)	-1.1	0.016	0.999

Glycolysis			

Phosphofructokinase, muscle (Pfkm)	2.1	1.00E-35	1.00E-35
Pyruvate kinase, muscle (Pkm2)	2.7	1.00E-35	1.00E-35
6-Phosphofructo-2-kinase/fructose-2,6-biphosphatase 3 (Pfkfb3)	2.9	1.19E-13	3.26E-09

Glycogenolysis			

Phosphorylase, glycogen, muscle (Pygm)	1.3	1.78E-14	4.88E-10
Phosphoglucomutase 1 (Pgm1)	2.8	1.00E-35	1.00E-35
Phosphoglucomutase 2-like 1 (Pgm2l1)	2.2	1.19E-13	3.25E-09

Fatty acid ß-oxidation			

Carnitine palmitoyltransferase 1b, muscle (CPT1b)	-1.4	3.52E-07	0.009
Hydroxyacyl-Coenzyme A dehydrogenase (Hadh)	-1.6	2.02E-07	0.005

Regulator genes (muscle fiber-type switch)			

SRY (sex-determining region Y)-box 6 (Sox6)	5.2	1.00E-35	1.00E-35
Myogenic differentiation 1 (Myod1)	4.4	1.11E-16	3.06E-12
Myogenin (Myog)	-3.7	1.78E-09	4.82E-05
Regulator of calcineurin 1 (Rcan1)	-4.6	3.51E-13	9.61E-09
SIX homeobox 1 (Six1)	2.0	5.68E-12	1.55E-07
SIX homeobox 2 (Six2)	5.0	9.39E-13	2.57E-08
Myostatin (Mstn)	4.4	3.74E-13	1.03E-08
Estrogen-related receptor γ (Esrrg)	-2.1	1.07E-10	2.91E-06
NFAT activating protein with ITAM motif 1 (Nfam1)	-1.7	3.34E-08	8.96E-04

The fold-change (FC) value indicates the relative amounts of transcripts in rats treated with zidovudine, compared with control animals. The Benjamini Hochberg *q *value indicates the false-discovery rate.

### Regulation of fiber composition

To elicit regulatory mechanisms of fiber transformation, we focused on transcription factors involved in muscle differentiation. In zidovudine-treated muscle, *MyoD*, which is expressed mainly in type II fibers [30], was upregulated (+4.41-fold; *P *= 1.01E-16), and the myogenic regulatory factor (MRF) transcribed from *myogenin *[31]), which is normally expressed predominantly in type I fibers, was downregulated (-3.75-fold; *P *= 1.78E-09) [32].

The homeodomain transcription factors *Six1 *and *Six2*, the transcriptional repressor *Sox6*, and the transcriptional coactivator *Eya1 *promote a switch from slow to fast fibers [33,34]. We found *Six1 *(+2.05-fold; *P *= 5.68E-12), *Six2 *(+5.00-fold; *P *= 9.39E-13), *Eya1 *(+1.42-fold; *P *= 8.09E-06), and *Sox6 *(+5.20-fold; *P *= 1.00E-35) upregulated in zidovudine-treated rat soleus muscle.

Estrogen-related receptor (ERR) γ enhances mitochondrial biogenesis and function, mtDNA content and the expression of contractile proteins specific to slow muscle and is physiologically highly expressed in soleus muscle [35]. We found *ERRγ *downregulated in zidovudine-exposed soleus muscle compared with untreated controls (-2.13-fold; *P *= 1.07E-10).

Thus, mitochondrial dysfunction is associated with a coordinate regulation of a multitude of transcription factors that orchestrate the transformation from type I to type II fibers.

## Discussion

The present study demonstrates a previously undescribed skeletal muscle fiber-type transformation from slow fibers to fast fibers in a mitochondrial myopathy. The changes in fiber-type composition occur in the absence of muscle regeneration and not only are demonstrated at the level of myosin heavy-chain isoforms and isoforms of other contractile proteins, but also are paralleled by adjustments in the metabolic profile and a switch from an oxidative to a more-glycolytic transcriptosome. From a mechanistic perspective, this response of muscle energetics to the primary defect in respiratory chain function may maintain muscle strength via increased recruitment of glycolysis for ATP production, at the expense of increased energetic cost. The switch to more-glycolytic type II fibers, which are characterized by an increased lactate production compared with type I fibers, could contribute to the hyperlactatemia observed in patients with mitochondriopathies [36]. The fact that hyperlactatemia is typically observed only, or at least is aggravated during exercise in patients with inherited mutations in mtDNA [37,38] can explain the normal lactate levels in our rats in whom blood was collected at rest. Fiber-type switching is also observed in conditions associated with impaired blood oxygenation [39] and diminished muscle microcirculation [40].

In COPD, muscle hypoxia is associated with an increased proportion of type II fibers [39,41], a reduced number of mitochondria [42], increases in glycolytic enzyme activity, and an impairment of oxidative capacity [43].

Patients with idiopathic inflammatory myopathies also reveal an increased proportion of fast fibers and a lower proportion of slow fibers compared with healthy controls [40]. Even in healthy humans exposed to high altitude, the proportion of type I fibers is decreased [44,45]. Although we failed to identify a single master switch of type I to type II fiber transformation, these observations indicate that an impairment of mitochondrial respiration of many causes promotes type II fiber formation.

Interestingly, slow fibers showed even less mtDNA content than did fast fibers in zidovudine-treated rats. This observation may be explained by the dynamics of the system (for example, the possibility that these slow fibers could still be in the process of converting). Alternatively, this finding could be explained with a physiologically higher mtDNA turnover in slow (oxidative) fibers compared with fast (glycolytic) fibers, and therefore an increased susceptibility to the inhibition of mtDNA replication conferred by zidovudine. Clearly, regulators of fiber type exist in addition to mtDNA content, and vice versa. Downregulation of the ERRγ may explain some of the biologic processes observed in our model, as ERRγ physiologically promotes a switch to slow muscle fibers and induces oxidative metabolism by increasing mitochondrial number, size, and functions [35].

Type II fibers in our study had a higher degree of atrophy than did type I fibers, despite the fact that the former appeared to be less dependent on mtDNA replication than the latter, as evidenced by a lesser degree of mtDNA depletion. Because muscle disuse affects mainly type I fiber diameters [46,47], the type II fiber atrophy in our model suggests a mechanism related to mitochondrial dysfunction. This hypothesis is further supported by the predominant type II fiber atrophy in other conditions associated with muscle hypoxia, such as COPD [41], systemic sclerosis [2-4,48], and inflammatory myopathies [5]. Age-related sarcopenia is also associated with a predominant atrophy of type II fibers and an increased abundance of fast myosin heavy-chain isoforms in soleus muscle [49]. It is interesting to speculate, whether mitochondrial dysfunction, which has also been implicated in aging, may be a driver of these characteristics of the aging muscle [50]. Myostatin has been described as a potent negative regulator of muscle mass, and increased myostatin expression is particularly associated with type II atrophy [51]. Consistent with this, we found a fourfold enhancement of myostatin transcription (Table 2). Sarcopenia, in combination with the disabled aerobic energy supply of slow-twitch fibers, can also explain muscle weakness on static and dynamic exercise, fatigue and muscle atrophy observed in patients with mitochondrial myopathies [52].

The effects of mitochondrial dysfunction and hypoxia on fiber-type composition have important clinical implications for training and rehabilitations programs by suggesting that exercise intolerance in mitochondrial dysfunction may be improved not only by cardiopulmonary mechanisms, but also by promoting fiber type II formation, either by resistance training, or pharmacologically by targeting the calcineurin-dependent nuclear factor of activated T-cells (NFAT) with calcineurin inhibitors [53,54].

In mitochondrial myopathies, muscle strength and oxidative capacity were improved without type I fiber enhancement [55,56], and in COPD, muscle strength and oxidative capacity were enhanced without alterations in lung function [41,57]. In idiopathic inflammatory myopathy, however, endurance training increased type I fiber proportions and diameters [40]. This difference could be explained by the preservation of mitochondrial function in idiopathic inflammatory myopathy, which enables type I fiber formation, and the impairment of mitochondrial function in inherited or acquired defects of the mitochondrial genome, which disables type I fiber formation. Clearly, more research is needed about the different effects of training programs on cardiopulmonary function, skeletal muscle microcirculation, oxidative capacity, and fiber-type composition in these different conditions.

## Conclusions

Our work demonstrates a type I to type II fiber transformation in a mitochondrial myopathy and a preferential atrophy in type II fibers. The skeletal muscle fiber-type transformation in the absence of fiber-type regeneration and observed adjustments from oxidative to glycolytic metabolism provide evidence for mitochondrial function as a new regulator of skeletal muscle fiber type and other metabolic capacities. The effects of mitochondrial dysfunction on fiber-type composition have important clinical implications for training and rehabilitations programs.

## Abbreviations

COPD: chronic obstructive pulmonary disease; COX: cytochrome *c *oxidase; ERR: estrogen-related receptor; IQR: interquartile ranges; MHC: myosin heavy chain; MRF: myogenic regulatory factor; mtDNA: mitochondrial DNA; NADH-DH: nicotinamide adenine dinucleotide hydrogen dehydrogenase; nDNA: nuclear DNA; SD: standard deviation; SDH: succinate dehydrogenase.

## Competing interests

The authors have no competing interests.

## Authors' contributions

DL and ACV carried out the experiments, participated in the design of the study and participated in writing the manuscript. DP performed the microarray analyses. NV and UAW conceived the study, participated in its design and coordination, analyzed the data, and wrote the manuscript. JBK participated in muscle-fiber analyses. EB carried out the blood analyses. All authors read and approved the final manuscript.

## Acknowledgements

This work was supported by DFG grant VE 492/2-1. The article-processing charge was funded by the German Research Foundation (DFG) and the Albert Ludwigs University Freiburg in the funding program Open Access Publishing. We also thank Karin Sutter and Carmen Kopp for expert technical assistance.
